# Prognostic Exploration of All-Cause Death in Gingival Squamous Cell Carcinoma: A Retrospective Analysis of 2076 Patients

**DOI:** 10.1155/2021/6676587

**Published:** 2021-03-26

**Authors:** Shuai Zheng, Jin Yang, Chengzhuo Li, Didi Han, Fengshuo Xu, Rahel Elishilia Kaaya, Wang ShengPeng, Jun Lyu

**Affiliations:** ^1^Department of Clinical Research, The First Affiliated Hospital of Jinan University, Guangzhou 510630, Guangdong, China; ^2^School of Public Health, Shaanxi University of Chinese Medicine, Xianyang, Shaanxi, China; ^3^School of Public Health, Xi'an Jiaotong University Health Science Center, Xi'an, Shaanxi, China; ^4^Cardiovascular Research Center, School of Basic Medical Sciences, Xi'an Jiaotong University Health Science Center, Xi'an, Shaanxi, China; ^5^Key Laboratory of Environment and Genes Related to Diseases of Ministry of Education, Xi'an Jiaotong University Health Science Center, Xi'an, Shaanxi, China

## Abstract

**Background:**

We aimed to establish a prognostic model for gingival squamous cell carcinoma (GSCC) that was superior to traditional AJCC staging and to perform a comprehensive comparison of the newly established nomogram with the AJCC staging system.

**Methods:**

We extracted 2,076 patients with gingival squamous cell carcinoma who had been entered into the SEER (Surveillance, Epidemiology, and End Results) database between 2004 and 2015, and randomly divided 70% of them into the training cohort and the other 30% into the validation cohort. Cox regression analysis was performed in combination with clinical experience and age, race, sex, marital status, tumor location, histological subtype, tumor grade, AJCC stage, chemotherapy status, radiotherapy status, and surgery status as possible prognostic factors. We evaluated and compared the two cohorts using the consistency index (C-index), area under the receiver operating characteristic curves, calibration curves, discriminant improvement index, and decision-curve analysis.

**Results:**

The Cox retrospective analysis showed that age, AJCC stage, tumor grade, histological subtype, radiotherapy status, and surgery status were significant factors to include in the new model of gingival squamous cell carcinoma. The other indicators were also better for the new model than for the AJCC staging system.

**Conclusion:**

We have developed and validated a nomogram for performing reliable gingival squamous cell carcinoma prognoses. The prognostic value of the nomogram is higher than that of the AJCC staging system. We expect that the inclusion of more-comprehensive and authoritative data (i.e., not just limited to residents of the United States) would also allow the construction of reliable nomograms for other populations.

## 1. Introduction

Oral cancer includes cancer of the lips, tongue, and mouth [tenth revision of the International Classification of Diseases (ICD-10) code C01–06], and the threat of oral cancer is becoming more serious in many parts of the world, particularly Europe. There were 45,780 new cases of oral and pharyngeal cancer reported in the United States in 2015, of which 8,650 were related to cancer [1]. Combined oral and oropharyngeal cancer (ICD-10 codes C01–06, C09-10, and C14) has become the sixth most common cancer worldwide, with its incidence being highest in Latin America, the Pacific, South Asia, Eastern Europe, and Southern Europe [3]. This situation has prompted a considerable amount of research into oral cancer, and now many research results are available in literature sources such as journals and magazines. Gingival cancer is a type of oral cancer, but it is often rarely analyzed or studied specifically.

Gingival cancer is a relatively rare malignant tumor that accounts for less than 10% of all oral cancers in Europe and the United States [4–6]. The incidence of gingival cancer is very low in many Western countries, while its incidence is much higher in China than in Europe and the United States, being second only to that of tongue cancer, one of the other oral cancers [7–9]. A study of oral cancer in China analyzed 3,377 patients diagnosed with oral cancer from 1964 to 2012 in a single hospital [9]. The largest proportion of the patients had tongue cancer (57.2%), followed by gingival cancer (13.3%) and then cancers of the buccal mucosa (8.1%), tongue base (8.0%), lips (4.8%), and posterior molar area (0.7%). Gingival cancer represents an enormous threat to health in many countries worldwide, and especially China [8].

Gingival squamous cell carcinoma (GSCC) is generally characterized by an exogenous mass whose main form is granular, papillary, and herniated [10]. Because the appearance of GSCC is atypical, being very similar to common periodontal lesions, it may be misdiagnosed by the general dentist during a clinical examination [11]. The current standard treatment for gingival cancer mainly involves surgery and radiotherapy, with the best method being surgery alone or comprehensive surgery-based treatment. Studies have shown that the long-term survival is inferior for simple chemoradiotherapy without surgery compared to that for surgical treatment [9]. Although there have been major developments in diagnosis and treatment during the past 4 decades, there has not been a major breakthrough in the treatment rate—the overall 5-year survival rate of oral squamous cell carcinoma (OSCC) has remained relatively stable at around 50% [12].

We are committed to making research contributions to improving the prognosis of GSCC. The TNM (tumor, node, and metastasis) stage classification system of the American Joint Committee on Cancer (AJCC), hereinafter referred to as the AJCC staging system, is the most commonly used staging systems in clinical practice and is currently the gold standard for the diagnosis of gingival cancer [10]. The present study took the different approach of establishing a comprehensive prognostic evaluation system for GSCC based on a nomogram, which is a mathematical model for predicting specific endpoints that combines multiple important factors. Nomograms have evolved into reliable and convenient risk quantification tools that have been widely used in prognostic assessments of various types of cancer. A well-defined nomogram helps doctors make the right choices in clinical decision-making and patient prognoses [13]. We screened and identified patients with GSCC registered in the Surveillance, Epidemiology, and End Results (SEER) database between 2004 and 2015 and created a nomogram containing multiple variables based on an analysis of the identified data.

## 2. Research Objects and Data

### 2.1. Data Source

To reduce the burden of cancer in the population, the American Cancer Institute established a database of epidemiological and final results (the SEER database) for cancer patients in the country in 1973. This is one of the most-representative large tumor databases in North America that contains incidence, prevalence, mortality, and other evidence-based medical information about cancer patients in the country over decades [14]. The use of SEER data does not require informed consent to be obtained from patients, and the SEER Cancer Registry does not provide case identification information.

### 2.2. Patient and Variable Selection

We used SEER^∗^ Stat software (version 8.3.5, https://seer.cancer.gov/) to screen patient data in the latest version of the SEER database (covering 18 registries). We used the following histology subtype codes in the third revision of the International Classification of Diseases for Oncology (ICD-O-3): 8070/2 (squamous cell carcinoma in situ), 8070/3 (squamous cell carcinoma), 8070/6 (squamous cell carcinoma, metastatic), 8071/3 (squamous cell carcinoma, keratinizing), 8072/3 (squamous cell carcinoma, large cell), 8073/3 (squamous cell carcinoma, small cell, nonkeratinizing), 8074/3 (squamous cell carcinoma, spindle cell), 8075/3 (squamous cell carcinoma, adenoid), 8076/2 (squamous cell carcinoma in situ with questionable stromal invasion), 8076/3 (squamous cell carcinoma, microinvasive), 8077/2 (squamous intraepithelial neoplasia, grade III), and 8078/3 (squamous cell carcinoma with horn formation).

**Figure 1:**
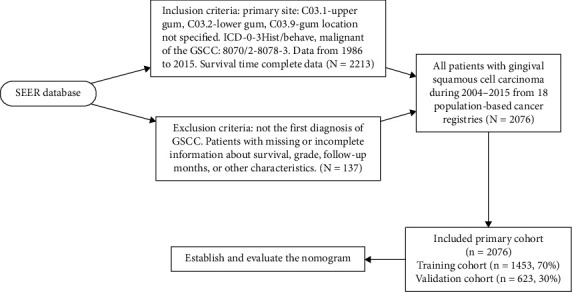
Flowchart of sample selection.

Based on our own clinical experience and patient interests, the variables we recorded were age, race, sex, marital status, histological subtype, tumor grade, AJCC stage, chemotherapy status, radiotherapy status, surgery status, and primary tumor site divided into upper gum (ICD-10 code C03.1), lower gum (ICD-10 code C03.2), and gum location not specified (ICD-10 code C03.9). We applied the sixth edition of the AJCC staging system, in which the surgery status, radiotherapy status, and chemotherapy status in the treatment program were divided into “yes” and “no/unknown.” We extracted 2,076 cases of patients with squamous cell carcinoma of the gingiva and excluded those who were unknown or incomplete cases or not confirmed by microscopy or autopsy; the specific exclusion process is shown in Figure 1.

### 2.3. Statistical Analysis and Establishment of the Nomogram

To construct and validate the nomogram, we randomly assigned 70% of the patients to the training cohort (*n* = 1,453) and 30% of the patients to the validation cohort (*n* = 623). A normality test was applied to the variables using a skewness/kurtosis test, which were then expressed as mean and standard-deviation or median and interquartile-range values depending on the distribution identified. The categorical variables were expressed as percentages. The multivariate Cox proportional-hazards regression model was used to identify survival-related factors, and the multivariate Cox regression model was used to analyze the survival-related factors we selected based on *P* < 0.1, which ultimately eliminated race, sex, marital status, and tumor grade as survival-related factors in this study. Based on the predictive model for determining prognostic factors, a nomogram was constructed to predict the 3-, 5-, and 8-year survival rates of patients with GSCC.

### 2.4. Verification and Evaluation of the Nomogram

Verifying and evaluating the performance of a nomogram involves the use of verification sets for identification and calibration. Receiver operating characteristic (ROC) curves were generated for testing the performance of the constructed nomogram based on the areas under the ROC curves (AUCs). The consistency between the predicted probabilities and actual results was evaluated in plots. The relative corrected consistency index (C-index) of the nomogram was also determined. In addition, the improvement in the prediction accuracy of the model was evaluated by calculating the relevant discriminant improvement index (IDI) [15]. Finally, we used decision-curve analysis (DCA) to assess the clinical usefulness and net effectiveness of the new predictive model [16].

All statistical analyses were performed using SPSS (version 24.0, SPSS, Chicago, IL) and R software (version 3.0.1; http://www.rproject.org). A probability value of *P* < 0.05 was considered statistically significant, and all tests were double-sided.

## 3. Results

### 3.1. Baseline Characteristics of the Study Population

The application of established inclusion and exclusion criteria yielded 2,076 patients who had been entered into the SEER database between 2004 and 2015. The survival time was available for all of the patients included in this study. R software was used to randomly divide the patients into the training and verification cohorts.  Table 1 presents their demographic characteristics, indicating that the proportions of males and females were roughly the same in the training cohort (51.7% and 48.3%, respectively) and the validation cohort (49.4% and 50.6%, respectively), as were the median ages at diagnosis (71 and 72 years, respectively). The proportions of Caucasians (84.2% in the training cohort and 84.1% in the validation cohort) were significantly higher than those of blacks and other races. In terms of pathological staging and clinical treatment, most of the patients were in AJCC stage IV, had tumor sites in the lower gingiva, and had the histological subtype of in situ squamous cell carcinoma rather than other types of squamous cell carcinoma (accounting for >70% of the patients). The vast majority of patients received surgery, with only a small proportion receiving radiotherapy. Chemotherapy does not provide confirmed positive clinical effects for GSCC, and so less than half of the patients received chemotherapy.

### 3.2. Prognostic Factors in Multivariate Cox Regression Analysis

The results of the multivariate Cox regression analysis were used to establish a multivariate model to identify independent prognostic variables. The results showed that sex, race, marital status, primary site, and chemotherapy status had no significant effects on the survival rate. Therefore, multivariate Cox regression analysis was performed with age, AJCC stage, tumor grade, histological subtype, radiotherapy status, and surgery status. Cox regression analysis showed that the significant parameters were the age of diagnosis [hazard ratio (HR) = 1.023, 95% confidence interval (CI) = 1.015–1.030, *P* ≤ 0.001], AJCC stage II (HR = 2.252, 95% CI = 1.608–3.153, *P* ≤ 0.001), AJCC stage III (HR = 3.052, 95% CI = 2.126–4.381, *P* ≤ 0.001), ICD-O-3 code 8072 (HR = 4.490, 95% CI = 1.948–10.347, *P* ≤ 0.001), radiotherapy (HR = 1.181, 95% CI = 0.974–1.432, *P* ≤ 0.1), surgery (HR = 3.489, 95% CI = 2.814–4.326, *P* ≤ 0.001), tumor grade II (HR = 1.552, 95% CI = 1.246–1.934, *P* ≤ 0.001), and tumor grade III (HR = 1.592, 95% CI = 1.203–2.106, *P* ≤ 0.01).  Table 2 lists the variables selected in the multivariate Cox regression analysis.

### 3.3. Nomogram Construction

**Figure 2:**
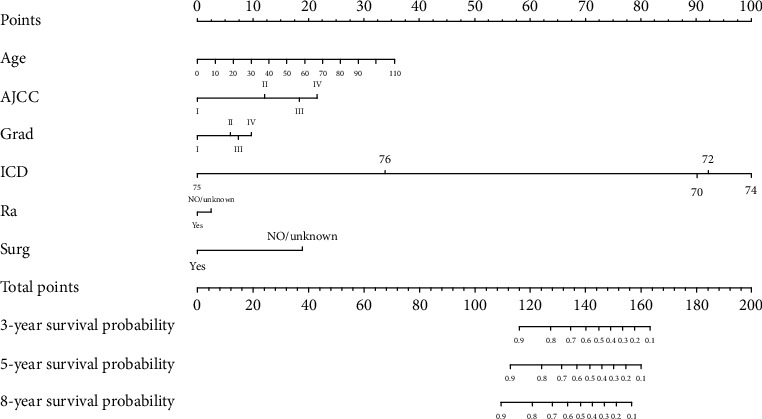
Nomogram of gingival squamous cell carcinoma.

A nomogram (Figure 2) was constructed based on the data for the logistic regression model in Table 2. The scale contains each significant predictor, and the nomogram is used to obtain the total score by adding the scores for each of the determined factors, which is then used to determine the predicted probability of surviving for 3, 5, and 8 years. It is easy to see from the nomogram that the protective factors include age <20 years, radiotherapy, and surgery. It is also intuitive to observe that cancer specific mortality is most affected by the subtype of gingival tumor, followed by age, AJCC staging, and surgery status, and less affected by the cell differentiation grade and radiotherapy status.

### 3.4. Verification of the Nomogram

**Figure 3:**
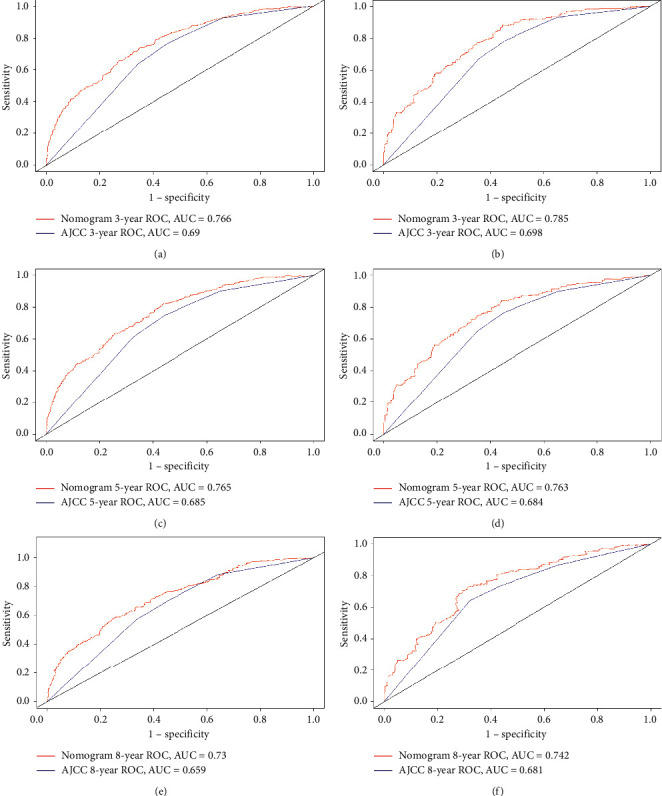
ROC curves between the nomogram and the AJCC staging system, training set (a, c, e), validation set (b, d, f).

The C-index is a very reliable indicator for evaluating the performance of a nomogram, with a higher C-index indicating better performance. The C-indexes for the training and validation cohorts were 0.660 and 0.663, respectively, for the AJCC staging system, while that for the nomogram was significantly higher, at 0.75.  Figure 3 provides a more intuitive comparison of ROC curves between the nomogram and the AJCC staging system. The AUCs for the nomogram and the AJCC staging system in the training cohort were 0.766 and 0.690, respectively, at 3 years, 0.765 and 0.685 at 5 years, and 0.730 and 0.659 at 8 years; the corresponding values in the validation cohort were 0.785, 0.698, 0.765, 0.684, 0.742, and 0.681, respectively. These findings clearly indicate that, regardless of the cohort or year, the AUC was higher for the nomogram than for the AJCC staging system.

**Figure 4:**
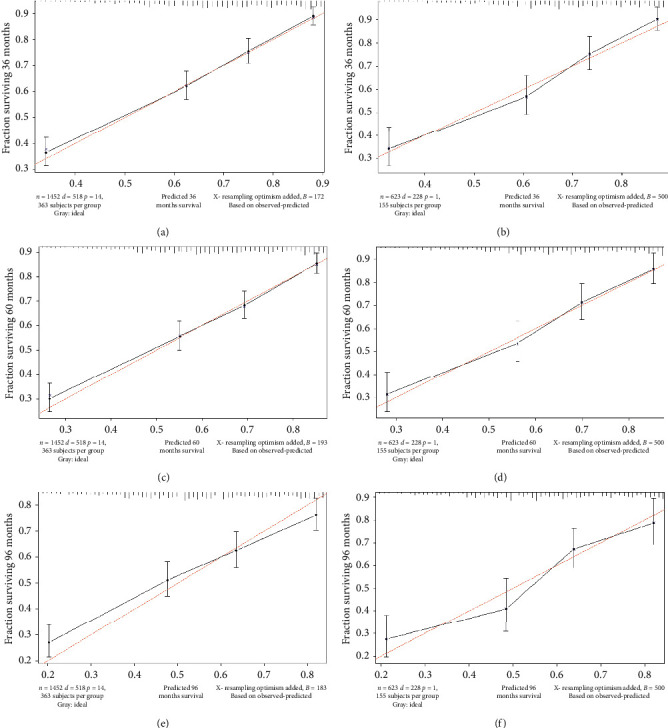
IDIs at 3, 5, and 8 years, training set (a, c, e), validation set (b, d, f).

IDI is a more sensitive indicator than the C-index. IDIs at 3, 5, and 8 years were 0.0902 (*P* ≤ 0.001), 0.0904 (*P* ≤ 0.001), and 0.08705 (*P* ≤ 0.001), respectively, in the training cohort, and 0.0682 (*P* ≤ 0.001), 0.0873 (*P* ≤ 0.001), and 0.0837 (*P* ≤ 0.001) in the validation cohort. We also used the new model to construct 3-, 5-, and 8-year calibration curves to analyze the consistency between the predicted probabilities and the observed outcomes.  Figure 4 shows that the new model is better than the old model.

**Figure 5:**
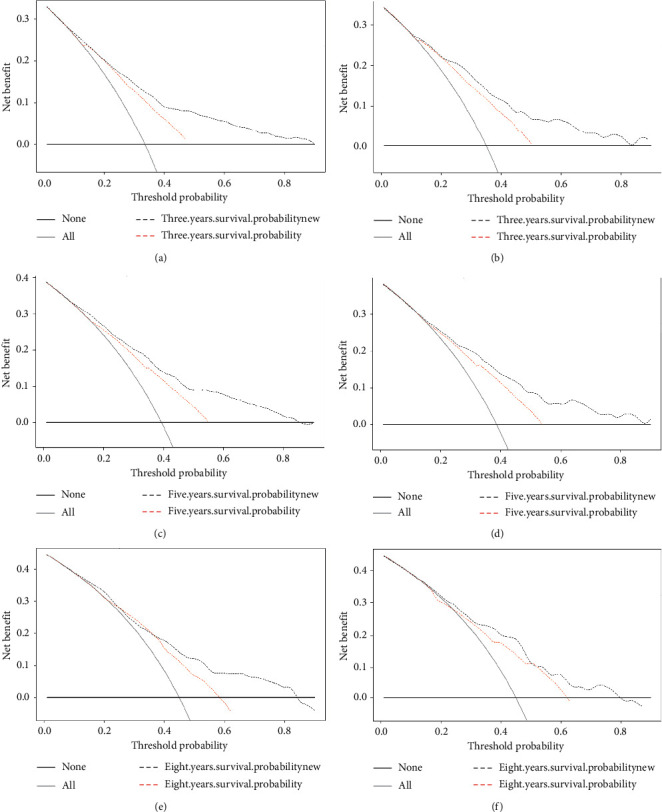
DCA curves for the 3, 5, and 8 years, training set (a, c, e), validation set (b, d, f).

DCA was used to evaluate the clinical usefulness of the nomogram.  Figure 5 shows that the curves for the 3-, 5-, and 8-year cancer specific survival predictions of the nomogram all appear above the corresponding curves for the traditional AJCC staging system, indicating the superior performance of the nomogram.

## 4. Discussion

The threat of oral cancer to human health means that more efforts need to be made in research, analysis, and interventions. GSCC is a unique type of malignant oral tumor whose clinical manifestations are similar to those of many other lesions, especially inflammatory lesions. Early GSCC usually appears in clinical practice as a white spot or erythema, and the minimal associated pain typically results in a diagnosis delay of 4–8 months [17]. The diversity of their clinical manifestations means that GSCCs are often misdiagnosed as benign tumors or other inflammatory reactions, which often delays the diagnosis and hence further worsens the outcome [11].

Gingival tumors often originate from keratinized mucosa, most commonly in the posterior mandibular fossa, which destroys the underlying skeletal structure and also causes tooth activity and shedding [18]. Surgery is the best treatment for gingival cancer. The choice of surgical method for mandibular gingival cancer depends on the stage of the disease while considering the removal of the mandible or the removal of the cervical lymph nodes. Because it is very close to the underlying bone and often invades the bone at an early stage, any tumor that invades the underlying bone is designated as T4 by the AJCC staging criteria, and so the surgical treatment of gingival cancer usually involves removal of the underlying bone [19]. For a small number of patients diagnosed with T1 lesions, the tumor can be completely removed by resecting the gingival mucosa. The necessity for marginal or mucosal resection can be determined based on the size of the tumor and whether the patient has teeth. Edge surgery is suitable when there is no basis for bone infiltration [20], and because a gingival tumor that is very close to the underlying periosteum and bone has a higher risk of metastasis [21], the 5-year survival rate is lower for GSCC than for other squamous cell carcinomas. Although many types of surgical resection are currently the most-effective means for treating gingival cancer [22], they can also cause serious functional problems such as difficulty in occlusion, chewing, excessive nasal congestion and rhinorrhea, and even oroantral fistulas [7]. The prognosis of GSCC remains a major problem. The most-important indicator of the prognosis is the clinical stage. If the tumor is small and localized, the 5-year cure rate is typically 60–70%, but the occurrence of cervical lymphatic metastasis reduces the survival rate to 25% [11].

GSCC is extremely prone to spreading, and hence a more-complete scientific diagnosis and treatment system is needed to prevent and treat GSCC. Despite improvements in diagnosis methods over the past 40 years, the 5-year survival rate of OSCC has remained relatively stable at around 50% [23]. Nomograms have been widely used in the prognosis of various cancers in recent years. Weiser et al. were the first to introduce a nomogram into the field of oncology, in 1998 [24]; since then, they have been used to predict the probability of various clinical prognostic events such as recurrence, metastasis, and death [25–27]. There is a considerable amount of evidence that nomograms are superior to universal staging systems—such as the AJCC staging system—in predicting the survival outcome of various solid tumors. Compared with AJCC, which can only partially estimate the clinical risk of cancer. The nomogram can use Cox regression to simplify the statistical prediction model to a continuous numerical estimate [24, 28–30] that accurately reflects the condition of each patient.

The purpose of this study was to establish a nomogram for GSCC. The SEER program has expanded to now cover 18 registration centers since its inception in 1973. More sites will become involved as technology advances, and currently the 18 geographically distinct regions included in the database account for approximately 30% of the United States population [14]. We screened 2,076 patients with GSCC from this representative database, and included age, race, sex, marital status, tumor location, histological subtype, tumor grade, AJCC stage, chemotherapy status, radiotherapy status, and surgery status as potential prognostic factors when multivariate Cox regression analysis was performed. Age, AJCC stage, tumor grade, histological subtype, radiotherapy status, and surgery status were finally identified as significant prognostic factors for GSCC. We used *P* < 0.1 as the standard for the multivariate Cox regression model because we wanted to avoid missing prognostic factors that affect GSCC (*P* < 0.1 for radiotherapy and *P* < 0.01 for tumor grade, *P* ≤ 0.001 for all other variables).

We compared the nomogram and the AJCC staging system using the C-index, calibration curve, IDI, and DCA. ROC curve analysis was used for evaluating the performance of the newly established nomogram, which provided relatively high C-indexes for the 3-, 5-, and 8-year survival rates in the training cohort (0.766, 0.765, and 0.730, respectively) and in the internal verification cohort (0.785, 0.765, and 0.742, respectively); the corresponding values for the AJCC staging system were 0.690, 0.685, 0.659, 0.698, 0.684, and 0.681, respectively. These findings indicate that our nomogram model fitted the randomly assigned training and validation cohorts well and performed better than the AJCC staging model.

We used calibration curves and IDI to assess the performance of the survival model in order to further determine whether the newly established prognostic model performed better and whether it should be used in clinical practice. The calibration curve describes the calibration of each model based on the consistency between the predicted probability and the observed results. A line close to 45 degrees in the calibration curve indicates that the nomogram predictions are well calibrated (Figure 3). IDI was used to analyze the overall improvement of the nomogram, and the results showed that the prediction abilities of the nomogram over 3, 5, and 8 years were 6.8%, 8.7%, and 8.4% better, respectively, than the AJCC staging system (all *P* ≤ 0.001). The positive results obtained when applying the nomogram further demonstrate its superior performance and good discriminating power. DCA is used to assess clinical usefulness, and it can show the minimum net benefit of adjusted scores in a combined index. Some studies have demonstrated the benefits of using DCA [30, 31] The current results show that the net benefit for the 3-, 5-, and 8-year DCA curves was greater for our model than for the traditional AJCC staging system (Figure 4). Therefore, our newly developed nomogram can be used to improve the predictive performance in GSCC compared to using AJCC staging system alone.

To the best of our knowledge, this study has yielded the first nomogram for predicting the 3-, 5-, and 8-year survival rates of GSCC patients. The analyses performed using the C-index, calibration mapping, IDI, and DCA support the use of the nomogram as a tool to help reorient and optimize treatment in this clinical setting.

## 5. Limitations

The data source for this study was the SEER database. Geographical factors meant that most of the included patients were Caucasian, and so the results are not applicable to all races, and there were also no data available on genetic mutations or deletions, obesity, or eating habits. Moreover, the data extracted from the SEER database are retrospective, and so there would be selection bias and inevitable confounding bias in the screened subjects. This means that the results obtained from the nomogram should only be utilized by clinicians. Further in-depth studies should include more-representative patients and more-comprehensive prognostic factors in attempts to produce a more-accurate prognostic model.

## 6. Conclusions

The increasing harm being done by GSCC to humans is prompting us to work hard to improve the control and treatment of this disease. This is the first time that a nomogram has been used to establish the prognosis in GSCC. Our model provides a greater clinical benefit than using the AJCC staging system. However, the data analyzed in this study apply to GSCC patients living in the United States, and so we hope to obtain more-comprehensive data in the future in order to further expand the clinical application of our nomogram.

## Figures and Tables

**Table 1:** Patient characteristics.

Variable	Training cohort	Validation cohort
Age at diagnosis	71 (61–80)	72 (62–81)
*Race n (%)*		
White	1223 (84.2)	530 (85.1)
Black	91 (6.3)	44 (7.1)
Other	138 (9.5)	49 (7.9)

*Sex n (%)*		
Male	750 (51.7)	308 (49.4)
Female	702 (48.3)	315 (50.6)

*Marital n (%)*		
Married	1183 (81.5)	507 (81.4)
Unmarried	269 (18.5)	116 (18.6)
Other		

*AJCC n (%)*		
I	362 (24.9)	156 (25.0)
II	271 (18.7)	111 (17.8)
III	183 (12.6)	66 (10.6)
IV	636 (43.8)	290 (46.5)

*Site n (%)*		
Lip upper	437 (30.1)	166 (26.6)
Lip lower	907 (62.5)	418 (67.1)
Other lip	108 (7.4)	39 (6.3)

*ICD n (%)*		
8070	1050 (72.3)	454 (72.9)
8071	375 (25.8)	157 (25.2)
8072	17 (1.2)	6 (1.0)
8074	8 (0.6)	4 (0.6)
8075	1 (0.1)	2 (0.3)
8076	1 (0.1)	

*Grade*		
I	440 (30.3)	182 (29.2)
II	767 (52.8)	340 (54.6)
III	242 (16.7)	99 (15.9)
IV	3 (0.2)	2 (0.3)

*Chemotherapy n (%)*		
Yes	302 (20.8)	115 (18.5)
No	1150 (79.2)	508 (81.5)

*Radiotherapy n (%)*		
Yes	694 (47.8)	294 (47.2)
No	758 (52.2)	329 (52.8

*Surgery n (%)*		
Yes	1256 (86.5)	546 (87.6)
No	196 (13.5)	77 (12.4)

**Table 2:** Selected variables by multivariate cox regression analysis.

Variable			
	HR^a^	95%CI	*P* value
Age at diagnosis	1.023	1.0151–1.030	≤0.001^*∗∗∗*^
*AJCC n (%)*			
I	Reference		
II	2.252	1.6080–3.153	≤0.001^*∗∗∗*^
III	3.052	2.1259–4.381	≤0.001^*∗∗∗*^
IV	4.196	3.0982–5.684	

*ICD n (%)*			
8070	Reference		
8071	1.108	0.9048–1.357	0.320955
8072	1.079	0.4794–2.427	0.85488
8074	4.49	1.9482–10.347	≤0.001^*∗∗∗*^
8075	3.26E-07	0.0000-inf	0.994676
8076	2.98E-06	0.0000-inf	0.991213

*Chemotherapy n (%)*			
Yes			
No			

*Radiotherapy n (%)*			
Yes	Reference		
No	1.181	0.9742–1.432	0.1.

*Surgery n (%)*			
Yes	Reference		
No	3.489	2.8139–4.326	≤0.001^*∗∗∗*^

*Grade*			
I	Reference		
II	1.552	1.2456–1.934	≤0.001^*∗∗∗*^
III	1.592	1.2028–2.106	≤0.001^*∗∗*^
IV	1.909	0.4596–7.930	0.373468

## Data Availability

SEER collects cancer incidence from population-based cancer registries with data coverage greater than 30% of the US population. The data we used is based on the November 2018 submission. We accessed these through the SEER^∗^ Stat software with additional approvals.

## Abbreviations

GSCC: Gingival squamous cell carcinoma
AJCC: American joint committee on cancer
CI: Confidence interval
SEER: Surveillance, Epidemiology, and End Results
C-index: Concordance index
AUC: Area under the time-dependent receiver operating characteristic curve
IDI: Integrated discrimination improvement
DCA: Decision-curve analysis.
